# Addressing Privacy Concerns in Sharing Viral Sequences and Minimum Contextual Data in a Public Repository During the COVID-19 Pandemic

**DOI:** 10.3389/fgene.2021.716541

**Published:** 2022-03-24

**Authors:** Lingqiao Song, Hanshi Liu, Fiona S. L Brinkman, Erin Gill, Emma J. Griffiths, William W. L Hsiao, Sarah Savić-Kallesøe, Sandrine Moreira, Gary Van Domselaar, Ma’n H. Zawati, Yann Joly

**Affiliations:** ^1^ Department of Human Genetics, Faculty of Medicine and Health Sciences, McGill University, Montreal, QC, Canada; ^2^ Faculty of Health Sciences, Simon Fraser University, Burnaby, BC, Canada; ^3^ British Columbia Centre for Disease Control, Vancouver, BC, Canada; ^4^ Institut National de Santé Publique du Québec, Québec, QC, Canada; ^5^ Public Health Agency of Canada (PHAC), Guelph, ON, Canada

**Keywords:** privacy, data-sharing strategy, health information access, contextual data, COVID-19, viral sequence, metadata, genomic (or scientific) governance

## Abstract

COVID-19 was declared to be a pandemic in March 2020 by the World Health Organization. Timely sharing of viral genomic sequencing data accompanied by a minimal set of contextual data is essential for informing regional, national, and international public health responses. Such contextual data is also necessary for developing, and improving clinical therapies and vaccines, and enhancing the scientific community’s understanding of the SARS-CoV-2 virus. The Canadian COVID-19 Genomics Network (CanCOGeN) was launched in April 2020 to coordinate and upscale existing genomics-based COVID-19 research and surveillance efforts. CanCOGeN is performing large-scale sequencing of both the genomes of SARS-CoV-2 virus samples (VirusSeq) and affected Canadians (HostSeq). This paper addresses the privacy concerns associated with sharing the viral sequence data with a pre-defined set of contextual data describing the sample source and case attribute of the sequence data in the Canadian context. Currently, the viral genome sequences are shared by provincial public health laboratories and their healthcare and academic partners, with the Canadian National Microbiology Laboratory and with publicly accessible databases. However, data sharing delays and the provision of incomplete contextual data often occur because publicly releasing such data triggers privacy and data governance concerns. The CanCOGeN Ethics and Governance Expert Working Group thus has investigated several privacy issues cited by CanCOGeN data providers/stewards. This paper addresses these privacy concerns and offers insights primarily in the Canadian context, although similar privacy considerations also exist in other jurisdictions. We maintain that sharing viral sequencing data and its limited associated contextual data in the public domain generally does not pose insurmountable privacy challenges. However, privacy risks associated with reidentification should be actively monitored due to advancements in reidentification methods and the evolving pandemic landscape. We also argue that during a global health emergency such as COVID-19, privacy should not be used as a blanket measure to prevent such genomic data sharing due to the significant benefits it provides towards public health responses and ongoing research activities.

## Context and Introduction

Accessible contextual data accompanying genomic sequence data are necessary for informed public health responses to emergencies such as COVID-19. As of May 2021, the COVID-19 pandemic has claimed the lives of over 22 thousand individuals in Canada alone (Public Health Agency of Canada, 2020). With global cases exceeding 140 million and an international death toll of over three million individuals, COVID-19 continues to be a public health emergency devastating the populations and economies of countries around the globe (John Hopkins Coronavirus Resource Center, 2020). While accelerated efforts in vaccine development and production hold significant promise (BBC News, 2020; CBC, 2020), it is evident that continued public health interventions will be needed to bring an “end” to the COVID-19 pandemic (Levin et al., 2020). Specifically, viral genomic data sharing by researchers and public health authorities will be crucial to informing ongoing local, provincial, national, and international public health responses (Walport and Brest, 2011; van Panhuis et al., 2014; Dye et al., 2016; Edelstein et al., 2018). For example, analyzing SARS-CoV-2 viral genomic sequences has been essential in elucidating transmission patterns, identifying variants with enhanced transmissibility or clinical severity, and the real-time analysis of outbreaks (Fang and Meng, 2020).

Beyond informing public health policy, rapidly depositing SARS-CoV-2 genomic sequences in open databases have been of fundamental importance for quickly developing COVID-19 vaccines, testing kits, and other research efforts. For example, the first SARS-CoV-2 genomic sequences deposited in the Global Initiative on Sharing Influenza (GISAID) database allowed for rapidly developing the Pfizer-BioNTech BNT162b2 vaccine candidate (Polack et al., 2020). Similarly, the SARS-CoV-2 sequences deposited in GISAID have also provided the basis for the accelerated development and deployment of numerous diagnostic testing kits (Bohn et al., 2020). Recently, the importance of COVID-based genomic data sharing has been increasingly underscored with the emergence of novel SARS-CoV-2 Variants of Concern (VOCs) (To et al., 2020; Mahase, 2021). The Canadian and international response to VOCs relies centrally on viral genomic sequencing to detect and track VOC transmission and to investigate key mutations that affect disease severity and the virus’s ability to escape natural and post-vaccination immunity (Volz et al., 2020). For example, the B.1.1.7 (Alpha), B.1.351 (Beta), and P.1 (Gamma) VOCs were all detected largely through a combination of epidemiological, contextual, and genomic data sharing (Volz et al., 2020; Mahase, 2021). This detection is hugely significant. Although it is impossible to fully quantify, failing to detect more virulent and or deadly VOCs in a timely manner would likely cause substantial delays in enacting the appropriate response measures (Davies et al., 2021).

Recognizing the promise of genomic data sharing, the Canadian COVID-19 Genomics Network (CanCOGeN) was launched to coordinate and upscale existing genomics-based research and surveillance efforts, with the goals of tracking viral introductions, informing the public health response, and exploring the relationship of viral and human genomes in individual outcomes (Genome Canada, 2021a). CanCOGeN is mandated to sequence up to 10,000 individuals (host) genomes and up to 150,000 viral sample genomes (Genome Canada, 2021b). The sometimes innately differing nature of data sharing in human genomics versus pathogen genomics elicits varying legal, ethical, governance, technological, and other practical concerns. Accordingly, the CanCOGeN project comprises of two main subgroups- CanCOGeN-HostSeq and CanCOGeN-VirusSeq to address topics specific to the individual and viral data sharing respectively, while overarching committees, such as the CanCOGeN Ethics and Governance, Implementation, and Coordination Committees also exist to synchronize the efforts of these two groups. As a part of its mandate, the Ethics and Governance Committee has been tasked with exploring the privacy and ethical concerns of sharing SARS-CoV-2 genomic sequences along with the relevant associated contextual data. Sequencing data alone provides little to no utility (Schriml et al., 2020). Interpreting sequence data alongside high-quality contextual data provides exponentially more meaningful findings. Descriptive data fields such as the date of sample collection, geographic region of origin, and the age of the individual are critical for the proper contextual interpretation of the sequencing data and analytical results when conducting genomic surveillance and investigating a broad range of research questions. In an effort to increase the utility of archived pathogen genomic data, using existing pathogen contextual data standards (MIxS and MIGS) and considering Canadian legislation, VirusSeq developed a concise list of 16 minimal contextual data fields (see Table 1) to be associated with deposited SARS-CoV-2 sequences.

**TABLE 1:** MIxS Compliance and Implementation Metadata Standards (Genomics Standards Consortium, 2021).

Field Name	Definition
sample collector sample ID	The user-defined name for the sample.
sample collected by	The name of the agency that collected the original sample.
sequence submitted by	The name of the agency that generated the sequence.
sample collection date	The date on which the sample was collected.
geo_loc_name (country)	The country where the sample was collected.
geo_) loc_name (state/province/territory)	The province/territory where the sample was collected.
organism	Taxonomic name of the organism.
Isolate	Identifier of the specific isolate.
isolation source	The material sampled (this information is encoded by 6 additional fields which need only be filled as applicable, depending on sample type; anatomical material, anatomical site, body product, environmental material, environmental site, collection device, collection method).
host (scientific name)	The taxonomic, or scientific name of the host.
host disease	The name of the disease experienced by the host.
host age	Age of host at the time of sampling.
host gender	The gender of the host at the time of sample collection.
sequencing instrument	The model of the sequencing instrument used.
consensus sequence software name	The name of software used to generate the consensus sequence.
consensus sequence software version	The version of the software used to generate the consensus sequence.

Despite the broadly accepted benefits of such data sharing towards both health policy and research, the CanCOGeN Ethics and Governance Committee has found that privacy and the protection of personal information are frequently stated as justifications to resist sharing minimal contextual data in direct association with the viral sequences they describe (Joly, 2020). Privacy as a challenge to data sharing is not exclusive to COVID-19 and has been well-documented (Butler, 2007; van Panhuis et al., 2014; Sorani et al., 2015; Bernier and Knoppers, 2020; Bonomi et al., 2020). In the current context, there are concerns that publicly archiving SARS-CoV-2 viral sequencing data in combination with the minimal set of contextual data will allow for the reidentification of individuals (Shean and Greninger, 2018; Joly, 2020). This paper reviews and addresses potential privacy risks of sharing pathogen sequencing data along with its accompanying minimum contextual data mainly under the Canadian legal context. However, many of the principles and reasoning used here can be similarly applied in an international context. The first section introduces the key concepts of identifiability and personal information. The second section discusses whether publicly sharing SARS-CoV-2 genomic sequences inherently threatens the privacy of individuals. The third section focuses on the privacy considerations of publicly archiving four (age, gender, province/territory of collection, and sample collection date) minimal contextual data fields associated with the viral sequences. The fourth section then discusses situations where the privacy risks are elevated in sharing specific fields of contextual data in certain contexts and outlines precautions that can be used to mitigate such risks. Finally, as a part of the deliberations of the VirusSeq Ethics and Governance Working Group, some concerns were raised regarding the risk of individual self-identification in publicly available formats. The final section addresses this point specifically and focuses on the question of whether the definition of “identifiability” includes self-identification.

### A Brief Review on the Definition of Personal Information and Its Relationship to Privacy

To assess the privacy risks of sharing viral sequencing data and its associated minimum contextual data, it is important to first address concerns as to whether such data constitutes “personal information,” which, in general, requires the individual’s consent or other justified reasons to share in the context of research (Office of the Privacy Commissioner of Canada, 2013). In Canada, with a federal-provincial division of powers, personal information is protected under numerous forms of federal and provincial privacy legislation (Bernier and Knoppers, 2020). At the national level, personal information collected by federal entities is subject to the *Privacy Act* (Privacy Act, 1985; Office of the Privacy Commissioner of Canada, 2019), while the *Personal Information Protection and Electronic Documents Act (PIPEDA)* applies to the personal information collected throughout the commercial sector (Office of the Privacy Commissioner of Canada, 2020; PIPEDA, 2000). Additionally, each province is entitled to enact its own privacy legislation, if such provincial legislation is considered “substantially similar” to PIPEDA (Office of the Privacy Commissioner of Canada, 2017). Indeed, there are numerous applicable laws in Canada. Despite this broad variety of laws governing the collection and disclosure of personal information in Canada, the definition of what constitutes “personal information” is relatively uniform, focusing on the feature of “identifiability.” For example, PIPEDA defines personal information as “information about an identifiable individual” (that is recorded in any form … ) (Office of the Privacy Commissioner of Canada, 2019; PIPEDA, 2000). Similarly, at the provincial level in Quebec, personal information is “information concerning a natural person that allows the person to be identified” (Act respecting Access to documents held by public bodies and the Protection of personal information, Québec, 1982). In British Columbia (BC), the BC *Personal Health Information Access and Protection of Privacy (E-Health) Act*, BC *Personal Information Protection Act*, and BC *Freedom of Information Protection of Privacy Act*, all hold similar definitions as those provided by the above laws (Freedom of Information and Protection of Privacy Act, British Columbia, 1996; Personal Information Protection Act, British Columbia, 2003; E-health (Personal Health Information Access And Protection of Privacy) Act British Columbia, 2008). Lastly, the Information and Privacy Commissioner of Ontario summarises that information is “personal” if “it is reasonable to identify an individual from the information (either alone or by combining it with other information)” (Information and Privacy Commissioner of Ontario, 2016b). Other countries around the globe have similarly emphasized the concept of “identifiability” in their privacy legislation. For example, the European Union’s *General Data Protection Regulation* (GDPR) states that personal information is “relating to an identified or identifiable natural person” (General Data Protection Regulation, 2016). In the United States, “personal health information” is designated individually identifiable information relating to the “(...) health status of an individual (...)” by the *Health Insurance Portability and Accountability Act* (HIPAA) (HIPPA, 1996). Similarly in China, personal information is defined as “information that can identify specific natural persons either by itself or when combined with other information and in Australia, the Australian *Privacy Act* also focuses on identifiability as a component of personal information (The Privacy Act, 1988; Civil Code of the People’s Republic of China, 2020). These numerous legal definitions across a wide variety of jurisdictions emphasize that identifiability is a necessary and ubiquitous requirement concerning the definition of personal information. As such, in evaluating the privacy risks of publicly archiving viral genomic data and its associated contextual data, it will be key to assess whether such data can be considered personal information. Here, we will focus on this question by discussing the potential identification risks of sharing SARS-CoV-2 viral genomic sequences and their associated contextual data.

### Does Publicly Archiving of SARS-CoV-2 Viral Sequences Inherently Create Privacy Risks?

While concerns regarding the privacy risks of certain contextual data fields have been raised, it seems intuitive to first consider whether SARS-CoV-2 viral genomic sequences alone generate any privacy risks. Is it possible for an individual to be identified through only publicly archived pathogen sequences? To consider this question, it is important to assess whether the SARS-CoV-2 viral genome can be used as an identifier. Viruses are frequently characterized by their “serial interval” and “mutation rate.” The serial interval describes the time between the onset of symptoms in an infector (individual that transmits the virus) individual and the infectee (individual infected by the virus from the infector), and with the SARS-CoV-2 virus, the serial interval is estimated to be close to 4 days (Du et al., 2020). While the mutation rate has been predicted to be once every 10–15 days (Duchene et al., 2020). Since the serial interval is shorter than the mutation rate, multiple infector-infectee pairs will likely share the same viral sequence. If different individuals are likely to share the same pathogen sequence, the pathogen sequence alone cannot be used to effectively distinguish between various sequenced individuals. It is also extremely unlikely that each tested individual would have a unique viral sequence, therefore it is equally improbable for SARS-CoV-2 sequences to pose a significant reidentification risk to the host. Moreover, if at the time of sequencing, an individual is found to be infected with a unique form of the virus, the mutation rate of the SARS-CoV-2 virus are such that if the individual were to be tested again in the future, they would be unlikely to possess the same viral sequence (Du et al., 2020; Duchene et al., 2020). Overall, it is extremely unlikely for SARS-CoV-2 sequences derived from an individual to be used as an effective identifier. Some have noted that it is possible for pathogen samples to be “contaminated” with human DNA. In this scenario, sharing viral sequencing data can be argued as possibly also sharing human genomic information (Population Health and Genomics Foundation, 2020). While possible, such risks are also very unlikely given that technical safeguards are routinely implemented to systematically and robustly subtract any human-like or non-viral sequences of all public-level viral sequence datasets (this task is often termed “de-hosting”) (Population Health and Genomics Foundation, 2020; Public Health Agency of Canada - National Microbiology Laboratory, 2021). De-hosting is a very common technique used to remove human-reads from pathogen sequence datasets. Tools used for de-hosting remove genomic reads that map onto to human reference genome and are well-validated. Applying such quality control and safety techniques ensure that the risk of reidentification from public-level viral sequencing data is extremely low. In summary, the innate characteristics of the SARS-CoV-2 virus are such that it is statistically unlikely for one-to-one unique host-to-pathogen matches to occur. Additionally, various computer-based techniques are employed to sufficiently remove human-like sequences from the viral sequences to further minimize reidentification risks before publicly archiving in any public database.

### Does the Minimum Contextual Data (List 1) CanCOGeN Intends to Pubicly Deposit Constitute “Personal Information” According to Canadian Privacy Legislation?

As previously mentioned, the utility of sequencing data from a public health or research perspective is often highly dependent on the thoroughness and quality of its accompanying contextual data (Schriml et al., 2020). Some typical examples of contextual data include “laboratory of origin, date of collection, individual age and gender, method of sampling, etc.” (Griffiths et al., 2020). Concerns have been raised that publicly releasing these data fields in association with the samples they describe could violate the privacy of individuals (Shean and Greninger, 2018; Joly, 2020). Here, the core question to assess is whether the minimal contextual data makes the associated pathogen data “identifiable” and is thus considered “personal information.” While the law often writes of identifiability in binary terms (i.e., an individual is either identifiable or non-identifiable), statistically speaking, identifiability is better conceived as a spectrum of probabilities. These probabilities range from 0 to 100%, where the percentage describes the certainty with which information can be attributed to a person (Rocher et al., 2019). As noted, oftentimes, the term “identifier” is used in this context to describe information that contributes to the reidentification or identification of an individual (Sweeney, 2000; Golle, 2006; Rocher et al., 2019). Many specific denominations of the term, such as “unique” identifier, “quasi-identifier”, or “direct” identifiers exist, all emphasizing their potential to increase the probability of personal identification. For example, a quasi-identifier refers to a combination of traits or attributes in a dataset that is not independently capable of identification, but when in combination with other accessible data, becomes highly identifying (Sweeney, 2000). Typical examples of quasi-identifiers include characteristics such as date of birth, gender, visible minority status, and profession (Sweeney, 2000).

While identifiability is not a simple binary nor a “yes” or “no” concept, few resources specifically address the question of when an individual statistically and quantitatively passes from the qualitative terms of “non-identified/non-identifiable” to “identified/identifiable.” Despite this, resources do exist. Echoing the stances of privacy researchers and data-release precedent, the Information and Privacy Commissioner of Ontario has published the *De-identification Guidelines for Structured Data*, a guide on the identifiability, privacy, and the release of data (Information and Privacy Commissioner of Ontario, 2016a). What is considered “identifiable” does not merely depend on the statistical probability of attribution, but rather it is also affected by the sensitivity (also sometimes referred to as the degree of the potential “invasion of privacy”) (Dyke et al., 2015; Information and Privacy Commissioner of Ontario, 2016a). The sensitivity of data considers the consequences to an individual if the privacy of such data were to be invaded. Some data is more sensitive because the contents it reveals are usually of greater consequence. For example, in general, the repercussions of revealing an individual’s psychiatric history are typically greater than revealing the same individual’s rhesus blood type (Dyke et al., 2015). For more sensitive data deemed to present a higher invasion of privacy, the criteria for what is considered identifiable is stricter. What is considered non-identifiable for information with low sensitivity can conversely be considered identifiable if such information were to be considered highly sensitive (Information and Privacy Commissioner of Ontario, 2016a). Ontario’s *De-identification Guidelines for Structured Data* defines a reidentification risk of below 5% to be considered acceptable for information with the potential for high sensitivity (a high invasion of privacy) (Information and Privacy Commissioner of Ontario, 2016a). In other words, if the combination of reasonably available information can “single out” 20 or fewer individuals from a pool of potential candidates, the individual who the information is about, should be considered “identifiable,” if the information is considered sensitive (Information and Privacy Commissioner of Ontario, 2016a). The smaller the pool of potential candidates, the more identifiable an individual is. Here, COVID-19 related testing data are considered more sensitive due to their revealing implications on an individual’s past or present health condition/status and past medical testing that they have undergone. In Canada, such health-based information is generally considered as sensitive if identifiable (Townsend v. Sun Life Financial, 2012). The de-identification guide thus recommends a threshold of 5% for high sensitivity data, 7.5% for medium, and 10% for low sensitivity data (Information and Privacy Commissioner of Ontario, 2016a).

In evaluating the potential privacy risks of openly depositing SARS-CoV-2 genomic sequences and their minimum contextual data, we are aware that the four data fields of 1) age (displayed in intervals of 10-years), 2) gender, 3) province/territory of collection, and 4) date of collection, are considered more problematic from a privacy and reidentification standpoint by various stakeholders (Sweeney, 2000; Golle, 2006; Rocher et al., 2019). The other 12 fields while useful for statistical analyses, do not appreciably impact the risk of reidentification (except in situations where these other fields indirectly act as an indirect proxy for one of these four fields, which will also be discussed). Therefore, we will primarily explore the privacy and reidentification risks of those four fields. As a reminder, the important primary consideration is whether these four data fields in combination with other “reasonably available” information can allow for the identification of an individual, and accordingly, whether the various privacy legislations of Canada and other jurisdictions are called into effect. Based on the most recently available census data available from each province and territory, and considering the three fields of age, gender, province/territory location, if the population were to be stratified by contextual data fields such as age and gender (note the data released by Stats Canada uses age intervals of 5 years instead of CanCOGeN Virus-Seq’s proposed 10-years intervals. The 5-years interval is more identifying, since a more specific age range will be inherently more identifying), the number of individuals in the majority of categories greatly exceeds 20 individuals (Statistics Canada, 2020a). This is true for even the most sparsely populated provinces/territories such as Prince Edward Island or Nunavut (Statistics Canada, 2020b; Statistics Canada, 2020c). This means that by using the contextual data identifiers of age category, province/territory, and gender, the vast majority of individuals are not considered identified to the threshold of 5%. In short, for most individuals in Canada, the three traits of province/territory, gender, and age do not constitute personal information, as they cannot be used to sufficiently identify an individual. Potential exceptions for this will be discussed in the next section. Lastly, the data-field “collection date” may appear to be a strong quasi-identifier for stratifying the population. Yet, this is not an accurate conceptualization of reidentification, as a reasonably competent third-party will not be able to link such information to the other contextual data fields. This is because the date that an individual is tested for COVID-19 cannot be information that is considered “reasonably available” (Townsend v. Sun Life Financial, 2012). A third-party individual cannot be expected to have access to an individual’s COVID-19 testing history (including date that the test was performed on) and to use this information in conjunction with the contextual field released in public databases to reidentify. In other words, the field of collection date cannot be used as an identifier (Sweeney, 2000; Golle, 2006; Rocher et al., 2019). Taken together, the four proposed contextual data fields should not be considered “personal information” and can be shared publicly. It is, however, important to note that identifiability is a contextual matter that sometimes exceeds factors such as identifiability and data sensitivity. There is a plethora of other factors such as the costs of identification, time available, the technology available, population pool, etc. that must also be considered (Beauvais, 2020). In some circumstances, certain data fields may disproportionately raise the risk of reidentification, for example, the field of “province” in low-population provinces such as Prince Edward Island (estimated pop. of 159,713 in 2020), and these cases will be discussed in the following section (Statistics Canada, 2020b).

### Situations Where Sharing the Sample’s Province of Origin, Gender, and Date of Collection May Disproportionately Increase the Risk of Identification

Identifiability is contextual and contingent on factors such as the population pool and confirmed cases in that specific province, and more (Information and Privacy Commissioner of Ontario, 2016a). This section discusses the reidentification risk in these scenarios. For provinces with a larger population, the risk of reidentification is inherently lower. The *Gordon* v. *Canada (Health)* 2008 federal court case established that the data field of “province” or “territory” can create a disproportionate risk of reidentification in provinces and territories with a smaller population (such as Prince Edward Island) (Gordon V. Canada (Health), 2008). Recognizing this, the CanCOGeN project has proposed to begin the data sharing process by replacing the “province” and “territory” field as “other” in all provinces/territories outside of British Columbia, Alberta, Ontario, and Quebec. The population, among other factors, in these four last provinces allow for the safe inclusion of this data field without appreciably raising the possibility of reidentification of such individuals. As a final note, data providers should be cautious about the level of geographic specificity they reveal when providing the methodologically relevant fields such as “collection agency.” For example, it is not uncommon for the collection agency to be the name of a local hospital, which then can reveal more detailed geographical location and increase the risk of reidentification. In short, measures should be taken so that information indicating an inappropriate level of geographic specificity is not provided.

Disclosing age and gender in conjunction with other fields can increase the risk of reidentification (Sweeney, 2000; Golle, 2006; Rocher et al., 2019). However, despite this increase, the ability to identify such individuals still falls below the previously mentioned threshold of 5% as already explained. However, it is important to note that the privacy risks of disclosing age are not uniform, as the number of very elderly or very young individuals make up a significantly smaller fraction of the population, and this should be considered (Statistics Canada, 2020a).

In some cases, provincial data report forms include non-traditional options for gender (e.g., non-binary and transgender) (CanCOGeN, 2021). Because individuals who do not conform to traditional binary terms make up a very small percentage of the population there is an increased risk of reidentification (Waite and Denier, 2019). Accordingly, VirusSeq has proposed to encompass all non-tradition gendered options into “non-disclosed” when publicly archived, consistent with what is done with the other initiatives (Statistics Canada, 2021). At the same time, such demographic information on non-binary individuals should still be collected as it contributes to equity, diversity, inclusion, and improves scientific representation of individuals and groups traditionally excluded from research (Bentley et al., 2017). These efforts will better ensure that the conducted research and their accompanying medical technical advances will represent marginalized individuals and groups as well as those who are traditionally well-represented. To reduce the potential privacy risks of this inclusion, this demographic data could be made available through controlled-access procedures.

The date of collection is another data field that originally had been thought to unacceptably increase privacy risks. Most of the current Health Canada diagnostic tests used for SARS-CoV-2 are based on Reverse Transcription polymerase Chain Reaction (RT-PCR), with results typically obtained 24–48 h after the date of sample collection (Health Canada, 2020 ). These delays considerably reduces the chances of associating the reported daily cases with the specific collection date. Furthermore, the typical range is not absolute, making it extremely unlikely to associate the testing date with the data release, as such, it will be equally unlikely for the collection date to be used as an identifier even if such information were to become public. In conjunction with what has already been written about the “reasonably available” standard, the date of collection does not appreciably increase the risk of reidentification. Notably, the introduction and mass dissemination of rapid COVID-19 testing kits, and potentially, other future advancements, may lead to the collection date and testing date being the same (Aguiar et al., 2020; Albert et al., 2021). If this were to unfold, and this date was disclosed with other identifying fields (e.g., province, when the province in question is “small”, gender, age, and the number of daily cases by province/neighbourhood), the risk of reidentification may increase. Although whether any increase makes a meaningful difference in terms of privacy is questionable and would also be case-dependent and contingent on multiple factors. Therefore, we recommend periodically monitoring reidentification risk to account for the increased efficiency of diagnostic methods, and other relevant developments that could potentially increase privacy risks.

### Does the Definition of “Identifiable” Include Self-Identification?

In the previous sections, we have emphasized that the concept of identifiability is an important component in the definition of “personal information.” Concerns regarding the risk of individual self-identification in publicly available formats have been raised. To be specific, if an individual is capable of identifying themself based on a list of contextual data and their viral genomic sequence in a public data repository or reported information, would that then mean that their information should be considered “identifiable” and cannot be shared publicly? The right to privacy is historically defined as being able to protect one’s personal life from intrusion by third parties (Warren and Louis, 1890). Similarly, in contemporary legislation, the concept of identifiability relates to identifiability from the perspective of an unauthorized third party and not that of an individual with access to high-level privy information. The emphasis on third parties is particularly important. The central notion proposed is that identifiability should be evaluated from the perspective of a third party, and not the individual themselves. This is confirmed by various precedents set by Canadian and European case-laws, best-practice documents, and peer reviewed literature guidelines which assess identifiability from a third person’s perspective. In the Canadian context, the 2008 *Gordon v. Canada (Health)* lawsuit, the federal courts considered the likelihood of individual reidentifiability specifically through the perspective of a third party attempting to reidentify an individual with access to information that is reasonably available (Gordon V. Canada (Health), 2008). More recently, in 2019, in the case *Canada (Information Commissioner) V. Canada (Public Safety and Emergency Preparedness) 2019*, the Federal courts once again assessed what constituted as “identifiable” and accordingly, the definition of what “personal information” is (Canada (Information Commissioner) v. Canada (Public Safety and Emergency Preparedness), 2019). Recall that the Canadian *Privacy Act* states that information is personal, “if there is a serious possibility that the information could be used to identify an individual either on its own or when combined with other available information.” In this case, the meaning of what “other available information” should mean was explored. The court reasoned, “the goal of the Privacy Act (…) is to prevent the undue disclosure of one’s personal information to others, not to oneself (…). That an individual might know that it is their name that is redacted from a document, for example, does not make the remainder of the document personal information.” (Canada (Information Commissioner) v. Canada (Public Safety and Emergency Preparedness), 2019). Similarly, in the EU Court of Justice, the issue of what constituted as personal information was once again considered through the perspective of a third party attempting to reidentify an individual (Patrick Bryer v. Bundesrepublik Deutschland, 2016). Likewise, the *Deidentification Guidelines for Structured Data* released by the Information and Privacy Commissioner of Ontario also evaluates and discusses the risks of reidentification from the perspective of either a “prosecutor” or “journalistic” third party (Information and Privacy Commissioner of Ontario, 2016a). Finally, in all scientific publications reviewed, identifiability is also always written in terms of an unauthorized third party (Sweeney, 2000; Golle, 2006; Rocher et al., 2019; Beauvais, 2020). The legal and logical basis of identifiability is always referred to from the perspective of an unauthorized third party with access to reasonably available information. The focus on third parties with respect to identifiability is justified given an individual’s knowledge of themselves and their personal information typically greatly exceeds that of any third party. A self-identification criterion would create a subjective, individually variable, and arbitrary standard to determine the exact definition and scope of personal information. In this sense, using a self-identification criterion would create an unnecessary, illogical, and inconsistent barrier to the free flow of information and ideas.

## Conclusion

Our paper presents the first attempt to analyze the privacy risks of sharing viral genomic sequences and their accompanying contextual data in the public domain, and this is likely relevant for many countries. The open disclosure of a minimal set of contextual data fields associated with the viral samples is crucial towards the timely promotion of research, collaboration, and scientific advancement in a time when it is desperately needed. We demonstrated using the Canadian privacy and public health framework that it is not contradictory to privacy laws to share a small amount of such data in association with genomic viral sequences. However, in certain scenarios when privacy risks may be disproportionately elevated, we also recommend considering special mitigating measures to significantly reduce risks. Measures such as disclosing age in intervals rather than the exact age and revealing the province/territory of origin only for Canadian provinces and territories with sufficiently large populations can be essential in ensuring the privacy of individuals. Despite our findings that legal privacy barriers are surmountable, concerns outside privacy are also appreciable. For example, despite an inability to sufficiently single out an individual, broad contextual information can still negatively implicate and stigmatize certain social groups or communities (Quigley, 2012). Although beyond the scope of this paper, issues beyond privacy must also be considered.

The COVID-19 pandemic has quickly evolved into a devastating global public health and economic crisis. In these circumstances, the free flow of low-privacy risk viral sequences and their associated contextual data is key in better understanding key factors surrounding COVID-19, from patience variability, transmission, to the creation of better testing, effective treatments, reliable vaccines, and beyond. Global public health emergencies should be understood by policymakers and privacy bodies as creating an imperative to review whether existing privacy laws offer sufficient flexibility to permit public health authorities and the research community to carry out their work for the public good. The Canadian Office of Privacy Commissioner declared that “during a public health crisis, privacy laws still apply, but they are not a barrier to appropriate information sharing.” Similar statements have also been made by other provincial privacy commissioners, including those of Alberta, Saskatchewan, and Ontario (Office of the Privacy Commissioner of Canada, 2020). Sharing SARS-CoV-2 genomic sequences alongside a minimal set of contextual data in the public domain with appropriate mitigating measures is, according to our findings, not contrary to the protection of personal information and privacy and is necessary for providing governments and researchers with the best available evidence to inform intervention. Our work mostly addresses concerns surrounding personal information and privacy. It does not explore the validity of arguments based on laws providing additional emergency powers to public health authorities in times of pandemics. It is our view that robust pathogen genomic surveillance should be facilitated in this day and age given the well-documented benefits in disease prevention and intervention responses (Grubaugh et al., 2019; Naveca et al., 2020). Indeed, while such data sharing is perhaps “beneficial” in regular times, in a global pandemic, data sharing ought to be characterized as both urgent and “necessary.”
